# Association of In-Ear Device Use With Communication Quality Among Individuals Wearing Personal Protective Equipment in a Simulated Operating Room

**DOI:** 10.1001/jamanetworkopen.2021.6857

**Published:** 2021-04-19

**Authors:** Don Luong Nguyen, Emily Kay-Rivest, Marc A. Tewfik, Michael Hier, Alexandre Lehmann

**Affiliations:** 1 Laboratory for Brain, Music and Sound Research (BRAMS), Centre for Research on Brain, Language or Music (CRBLM), Royal Victoria Hospital, Department of Otolaryngology–Head and Neck Surgery, McGill University, Montreal, Quebec, Canada; 2 Royal Victoria Hospital, Department of Otolaryngology–Head and Neck Surgery, McGill University, Montreal, Quebec, Canada; 3 Jewish General Hospital, Department of Otolaryngology–Head and Neck Surgery, McGill University, Montreal, Quebec, Canada

## Abstract

**Importance:**

The COVID-19 pandemic has brought forth new challenges for health care workers, such as the daily use of personal protective equipment, including reusable facial respirators. Poor communication while wearing respirators may have fatal complications for patients, and no solution has been proposed to date.

**Objective:**

To examine whether use of an in-ear communication device is associated with improved communication while wearing different personal protective equipment (N95 mask, half-face elastomeric respirator, and powered air-purifying respirator [PAPR]) in the operating room.

**Design, Setting, and Participants:**

This quality improvement study was conducted in June 2020. Surgical residents from the Department of Otolaryngology–Head and Neck Surgery at McGill University in Montreal, Quebec, Canada, were recruited. All participants had normal hearing, were fluent in English, and had access to the operating rooms at the Royal Victoria Hospital.

**Exposures:**

All participants performed the speech intelligibility tasks with and without an in-ear communication device.

**Main Outcomes and Measures:**

Speech intelligibility was measured using a word recognition task (Modified Rhyme Test) and a sentence recognition task (AzBio Sentence Test). A percentage correct score (0% to 100%) was obtained for each speech intelligibility test. Listening effort was assessed using the NASA Task Load Index. An overall workload score, ranging from 0 points (low workload) to 100 points (high workload), was obtained.

**Results:**

A total of 12 participants were included (mean [SD] age, 31.2 [1.9] years; 8 women [66.7%]). AzBio Sentence Test results revealed that, while wearing the N95 mask, the mean (SD) speech intelligibility was 98.8% (1.8%) without the in-ear device vs 94.3% (7.4%) with the device. While wearing the half-face elastomeric respirator, the mean speech intelligibility was 58.5% (12.4%) without the in-ear device vs 90.8% (8.9%) with the device. While wearing the PAPR, the mean speech intelligibility was 84.6% (9.8%) without the in-ear device vs 94.5% (5.5%) with the device. Use of the in-ear device was associated with a significant improvement in speech intelligibility while wearing the half-face elastomeric respirator (32.3%; 95% CI, 23.8%-40.7%; *P* < .001) and the PAPR (9.9%; 95% CI, 1.4%-18.3%; *P* = .01). Furthermore, use of the device was associated with decreased listening effort. The NASA Task Load Index results reveal that, while wearing the N95 mask, the mean (SD) overall workload score was 12.6 (10.6) points without the in-ear device vs 17.6 (9.2) points with the device. While wearing the half-face elastomeric respirator, the mean overall workload score was 67.7 (21.6) points without the in-ear device vs 29.3 (14.4) points with the in-ear device. While wearing the PAPR, the mean overall workload score was 42.2 (18.2) points without the in-ear device vs 23.8 (12.8) points with the in-ear device. Use of the in-ear device was associated with a significant decrease in overall workload score while wearing the half-face elastomeric respirator (38.4; 95% CI, 23.5-53.3; *P* < .001) and the PAPR (18.4; 95% CI, 0.4-36.4; *P* = .04).

**Conclusions and Relevance:**

This study found that among participants using facial respirators that impaired communication, a novel in-ear device was associated with improved communication and decreased listening effort. Such a device may be a feasible solution for protecting health care workers in the operating room while allowing them to communicate safely, especially during the COVID-19 pandemic.

## Key Points

**Question:**

Is an in-ear communication device associated with improved speech intelligibility among surgical residents using reusable respirators in the operating room?

**Findings:**

In this quality improvement study including 12 surgical residents, speech intelligibility was 90.8% with the use of an in-ear device compared with 58.5% without this device among participants wearing half-face elastomeric respirators. Use of the device was also associated with decreased listening effort.

**Meaning:**

Results of this study suggest that an in-ear communication device is a viable solution for restoring communication to a normal level while using personal protective equipment.

## Teaser

This quality improvement study assesses 6 listening conditions involving the use of an in-ear radio device and 3 types of facial personal protective equipment (PPE) in a simulated operating room to identify obstacles and solutions to better communication during the COVID-19 pandemic.

## Introduction

The COVID-19 pandemic has brought never-before-encountered challenges to health care. Among these challenges is the global shortage of personal protective equipment (PPE). The influx of large numbers of critically ill patients has led to the rapid depletion of N95 masks, which protect health care workers from contagious airborne particles. In a survey conducted by the American Nurses Association between July 24, 2020, and August 14, 2020, 1 in 3 nurses (out of 21 000 nurse respondents) reported ongoing N95 mask shortages.^1^ Moreover, 58% of these nurses reported being required to reuse single-use N95 masks for more than 5 days.^1^ One solution to this shortage is the use of industrial types of reusable facial respirators, such as half-face elastomeric respirators, full-face elastomeric respirators, and powered air-purifying respirators (PAPRs).

An unexpected consequence of using PPE is a substantial reduction in speech intelligibility. According to Radonovich et al,^2^ the odds of correctly hearing a word spoken by a health care worker who is wearing a half-face elastomeric respirator are 46%, which means that during an acute situation, an individual (including a health care worker) may understand only half of what is being said. According to the National Institute for Occupational Safety and Health, a respirator can be released only if its speech intelligibility performance is 70% or greater.^3^ Furthermore, the US military considers “normal acceptable intelligibility” as 98% of sentences that are heard correctly.^3^ Given the consequences of errors in communication in a military operation as well as in the operating room, the performance of these respirators must be closely examined.

Communication errors are associated with adverse events in the operating room.^4^ From 2004 to 2014, The Joint Commission evaluated more than 4000 adverse events in health care and found communication breakdown to be the most common factor in complications.^5^ Specifically, 70% of these adverse events were associated with communication failures, 75% of which resulted in patient death.^4^ Furthermore, beyond speech intelligibility, impaired communication can lead to an increased listening effort, which is the mental exertion required to attend to and understand an auditory message.^6^ A higher listening effort has been associated with workplace fatigue and burnout.^6^

As COVID-19 cases continue to surge, the use of reusable respirators will become more prevalent. Poor communication while wearing these masks could lead to potentially fatal complications for patients, and no solution has been proposed to date.

In this quality improvement study, we examined whether an in-ear communication device that was originally designed for heavy industrial settings is associated with improved communication while different PPE (N95 mask, half-face elastomeric respirator, and PAPR) is worn in the operating room. We hypothesized that the in-ear device would be able to restore to a near-normal level communication while using a respirator.

## Methods

### Participants and Procedures

We invited surgical residents from the Department of Otolaryngology–Head and Neck Surgery of McGill University to participate in this study on a voluntary basis. Eligible participants had normal hearing (defined as air conduction audiometric thresholds ≤25 dB HL at 0.5 to 4 kHz bilaterally), were fluent in the English language, and had authorization to access the operating rooms at the Royal Victoria Hospital in Montreal, Quebec, Canada. This quality improvement study, which was conducted in June 2020, was approved by the McGill University Health Centre Research Ethics Board. Written informed consent was obtained from all participants. We followed the Standards for Quality Improvement Reporting Excellence (SQUIRE) reporting guideline.

Hearing was screened in a quiet room using a portable audiometer (MA 25; MAICO Diagnostics). After screening, we conducted speech recognition testing in an operating theater. To simulate a listening environment that is similar to a live operation, we added ambient noise by turning on the suction machine and a warming blanket. The noise was present continuously throughout the experiment and was measured at the participant’s position at 60 dBA. In addition, participants were asked to perform a suturing task during the speech recognition test. Each participant acted as the listener and was paired with 1 of us (E.K.R. or D.L.N.), who acted as the talker. The listener and talker were positioned 1 m apart on either side of the surgical table. Participants were asked to repeat, to the best of their ability, the target words or target sentences spoken by the experimenter.

**Figure 1.**
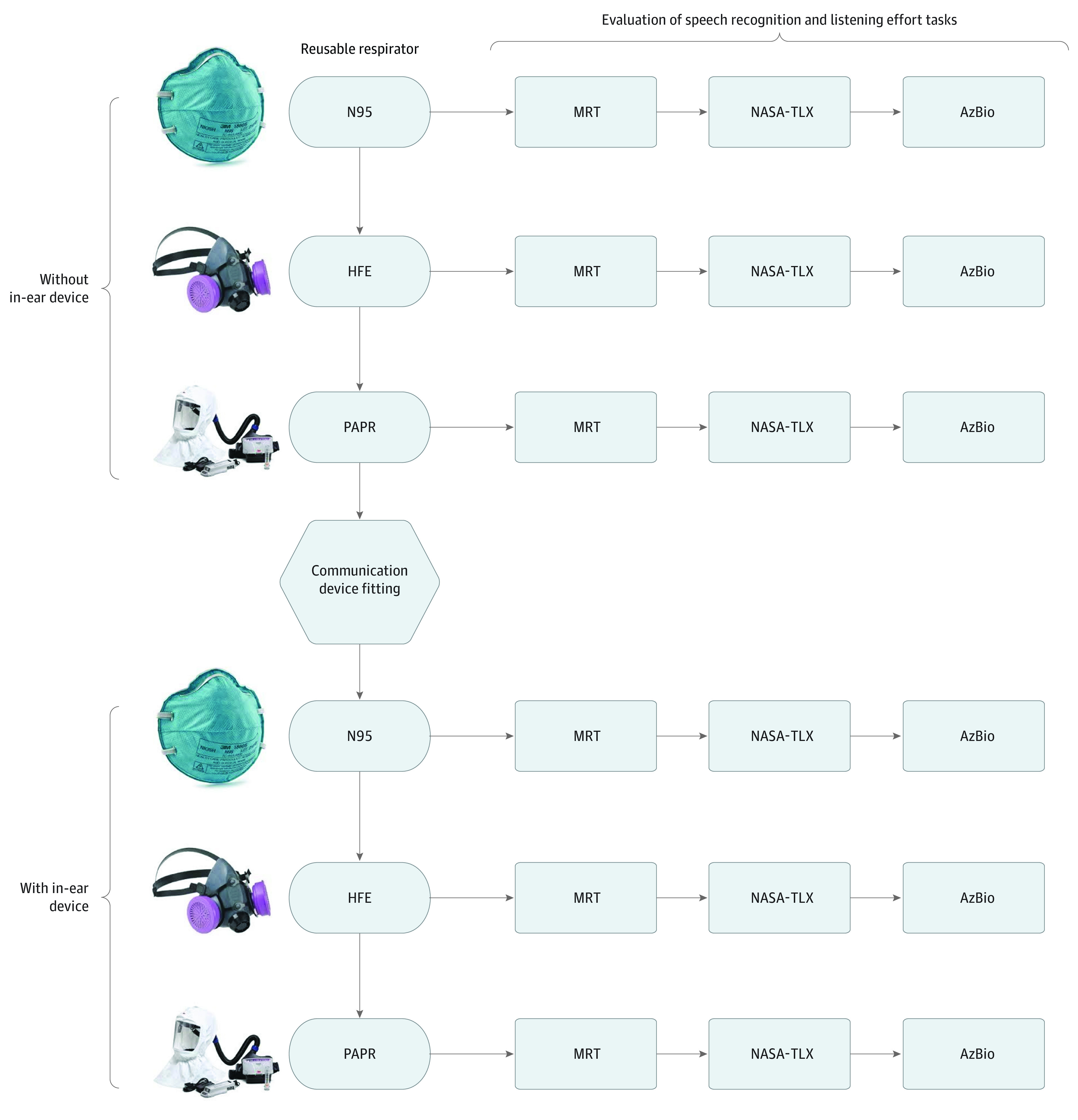
Testing Procedures AzBio indicates AzBio Sentence Test; HFE, half-face elastomeric respirator; MRT, Modified Rhyme Test; NASA-TLX, NASA Task Load Index; and PAPR, powered air-purifying respirator.

Speech recognition testing was performed with an in-ear device (aided) and without an in-ear device (unaided), whereas each talker wore 3 different types of PPE (N95 mask, half-face elastomeric respirator, and PAPR), presenting a total of 6 different listening conditions. Both the listener and the talker were fitted with the in-ear device in the aided condition (Figure 1). This device captured and transmitted the voice through a microphone in the ear canal rather than outside of the PPE.

### Speech Intelligibility and Listening Effort

For each listening condition, speech intelligibility was first assessed using speech material from the Modified Rhyme Test (MRT). The MRT is a standardized method for measuring the intelligibility of speech over communication systems and has been used in previous studies to evaluate speech intelligibility with respirators.^2^ The talker uttered each target word with the carrier phrase, “The word is….” The listener was instructed to repeat the word that was heard. A total of 50 six-word sets (1 MRT list) were tested for each listening condition, and the percentage correct score (0% to 100%) was calculated. Speech material for each participant was randomized using the same method described by Giguère et al.^7^ Briefly, the 50 sets were shuffled 4 times to create 4 different sequences. In total, 24 unique lists were created for this study.

Speech material from the AzBio Sentence Test was also used to measure speech intelligibility.^8^ The experimenter read each sentence in a conversational manner. The participant was asked to repeat as many words from the sentence as possible and was encouraged to use sentence context to fill in the blanks. A total of 20 sentences (1 AzBio Sentence Test list) were tested for each listening condition, and a percentage correct score (0% to 100%) was calculated for 1 list. Six AzBio Sentence Test lists, 1 per listening condition, were chosen at random from the available 15 lists for each participant.

Listening effort associated with the MRT task was measured using the NASA Task Load Index (NASA-TLX).^9^ Participants were asked (1) to rate six 100-point scales corresponding to different aspects of workload associated with the task and (2) to weigh each scale. An overall workload score for each listening condition, ranging from 0 (low workload) to 100 (high workload) points, was obtained by calculating the weighted mean.

### Communication Device and Personal Protective Equipment

The SonX (loaned to us by EERS Inc) was used as the communication device in this study. This in-ear radio device records the speaker’s voice in their ear canal and, through an innovative algorithm, transmits that voice clearly to other SonX users. It was originally designed for use with respirators in heavy industrial settings in which workers are exposed to dangerously loud noise and toxic fumes. For this study, the device was used in transparency mode to gain the benefits of the novel voice transmission method without losing auditory awareness of the environment. The output volume was fixed to the same level for all participants.

Three PPE types were used: N95 mask, half-face elastomeric respirator, and PAPR. The half-face elastomeric respirator included a filter cartridge and a surgical mask that covered the exhalation valve. Under the PAPR hood, a surgical mask was worn by the experimenter. The hood was connected to the air blower and was turned on during the test.

### Statistical Analysis

The speech intelligibility score and overall workload score were modeled using a linear mixed-effect model. The model included fixed effects for PPE (N95 mask, half-face elastomeric respirator, and PAPR), device (unaided or aided), and interaction of PPE and device. A random intercept was included to take into account the variability between participants. No imputation technique was used because the analysis model accounts for missing data.

The significance threshold was set at *P* = .05, and the *P* values reported were 2-sided. Analyses were performed with the lme4, lmerTest, and car packages in R (R Foundation for Statistical Computing). *F* and *P* values were calculated using the Kenward-Roger approximation of degrees of freedom. Post hoc pairwise comparisons were performed using the emmeans package in R. This analysis compared all pairs of PPE by device combinations to further investigate the differences between listening conditions (eTables 1-3 in the Supplement). The CIs and *P* values were adjusted for multiple comparison (*k* = 15) using the Sidak method.

## Results

This study included 12 participants, with a mean (SD) age of 31.2 (1.9) years. Eight participants (66.7%) were women, and 4 were men (33.3%). The main characteristics of participants are listed in Table 1. Some listening conditions could not be completed with participants 1 to 4 because of equipment malfunction. A summary of completed listening conditions for each participant can be found in eTable 4 in the Supplement.

**Table 1.** Demographic and Hearing Characteristics

Characteristic	Mean (SD)
No. of participants	12
Age, y	31.2 (1.9)
Sex, No. (%)	
Female	8 (66.7)
Male	4 (33.3)
Hearing status, dB HL^a^	
PTA right ear	14.0 (4.9)
PTA left ear	14.0 (5.2)

Abbreviation: PTA, pure-tone average.

^a^ Calculated as the 4-frequency PTA of 500, 1000, 2000, and 4000 Hz.

### Impaired Speech Intelligibility

Without an in-ear device, speech remained intelligible while wearing the N95 mask, whereas speech intelligibility was significantly lower with the half-face elastomeric respirator or the PAPR. As evaluated by the MRT, mean (SD) speech intelligibility was 75.4% (13.5%) while using the unaided PAPR, 48.8% (12.3%) while using the unaided half-face elastomeric respirator, and 93.8% (6%) while using the unaided N95 mask. As evaluated by the AzBio Sentence Test, mean (SD) speech intelligibility was 84.6% (9.8%) while using the unaided PAPR, 58.5% (12.4%) while using the unaided half-face elastomeric respirator, and 98.8% (1.8%) while using the unaided N95 mask vs 94.3% (7.4%) with the aided mask (Table 2).

**Table 2.** Results of Speech Intelligibility and Listening Effort Tests

Listening condition	Mean score (SD) [range], % correct		NASA-TLX workload score, mean (SD) [range], points
	MRT	AzBio	
Unaided: tested without in-ear device			
N95 mask	93.8 (6) [80-100]	98.8 (1.8) [94.2-100]	12.6 (10.6) [2-28.3]
PAPR	75.4 (13.5) [56-100]	84.6 (9.8) [69-98.5]	42.2 (18.2) [21.7-79.3]
Half-face elastomeric respirator	48.8 (12.3) [26-74]	58.5 (12.4) [24.1-71]	67.7 (21.6) [23-90.7]
Aided: tested with in-ear device			
N95 mask	92.7 (3.2) [88-98]	94.3 (7.4) [76.7-98.6]	17.6 (9.2) [6.3-33.3]
PAPR	90.4 (5.6) [82-100]	94.5 (5.5) [82.8-99.3]	23.8 (12.8) [6.7-48.7]
Half-face elastomeric respirator	86.5 (5.6) [76-96]	90.8 (8.9) [70.3-100]	29.3 (14.4) [8.3-54.7]

Abbreviations: AzBio, AzBio Sentence Test; MRT, Modified Rhyme Test; NASA-TLX, NASA Task Load Index; PAPR, powered air-purifying respirator.

**Figure 2.**
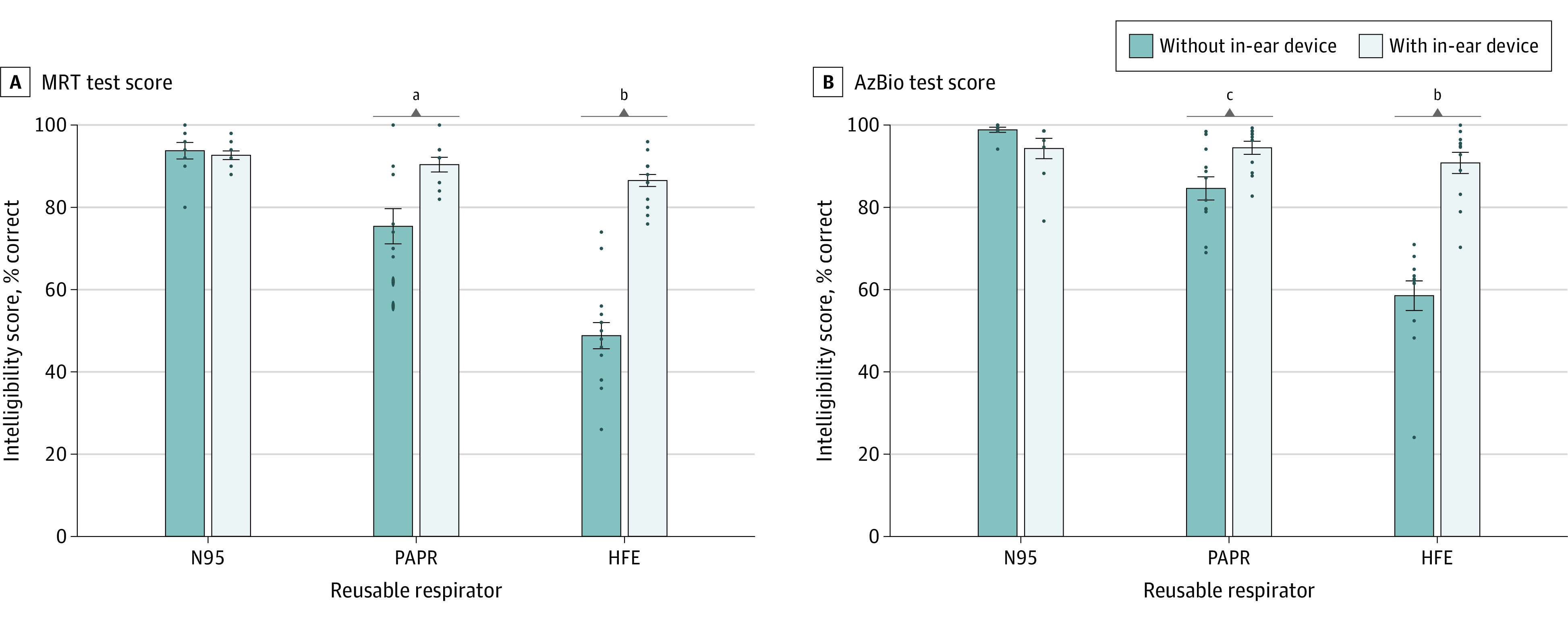
Association of In-Ear Device Use With Restored Speech Intelligibility While Wearing Reusable Respirators AzBio indicates AzBio Sentence Test; HFE, half-face elastomeric respirator; MRT, Modified Rhyme Test; and PAPR, powered air-purifying respirator. ^a^Significant at *P* = .01 threshold. ^b^Significant at *P* = .001 threshold. ^c^Significant at *P* = .05 threshold.

Compared with the unaided N95 mask, wearing the half-face elastomeric respirator was associated with a decrease in speech intelligibility of 44.4% (95% CI, 33.1%-55.7%; *P* < .001) as evaluated by the MRT (Figure 2A), and 40.8% (95% CI, 31.6%-50.1%; *P* < .001) as evaluated by the AzBio Sentence Test (Figure 2B). With the PAPR, speech intelligibility was lowered by 18.2% (95% CI, 6.1%-30.3%; *P* < .001) as evaluated by the MRT (Figure 2A) and by 14.8% (95% CI, 5.5%-24.0%; *P* < .001) as evaluated by the AzBio Sentence Test (Figure 2B).

### Restored Normal Communication 

Use of an in-ear device while wearing the half-face elastomeric respirator and the PAPR was associated with speech intelligibility levels that were comparable to levels observed with the unaided N95 mask, reflecting little to no degradation in speech intelligibility. As evaluated by the MRT, mean (SD) speech intelligibility was 75.4% (13.5%) while using the unaided PAPR vs 90.4% (5.6%) while using the aided PAPR, and was 48.8% (12.3%) while using the unaided half-face elastomeric respirator vs 86.5% (5.6%) while using the aided half-face elastomeric respirator. As evaluated by the AzBio Sentence Test, mean (SD) speech intelligibility was 84.6% (9.8%) while using the unaided PAPR vs 94.5% (5.5%) while using the aided PAPR, and 58.5% (12.4%) while using the unaided half-face elastomeric respirator vs 90.8% (8.9%) while using the aided half-face elastomeric respirator (Table 2).

Use of an in-ear device while wearing the PAPR was associated with a significant increase in speech intelligibility of 15.0% (95% CI, 3.3%-26.7%; *P* = .004) as evaluated by the MRT (Figure 2A) and 9.9% (95% CI, 1.4%-18.3%; *P* = .01) as evaluated by the AzBio Sentence Test (Figure 2B). An even larger increase in speech intelligibility was found with the half-face elastomeric respirator: 37.7% (95% CI, 28.1%-47.3%; *P* < .001) as evaluated by the MRT (Figure 2A) and 32.3% (95% CI, 23.8%-40.7%; *P* < .001) as evaluated by the AzBio Sentence Test (Figure 2B).

Speech intelligibility with the aided PAPR was not significantly different from that observed with the unaided N95 mask: 3.2% difference (95% CI, 0%-15.3%; *P* > .99) as evaluated by the MRT (Figure 2A) and 4.9% difference (95% CI, 0%-14.2%; *P* = .82) as evaluated by the AzBio Sentence Test (Figure 2B). Similar results in speech intelligibility were found when comparing the aided half-face elastomeric respirator with the unaided N95 mask: 6.7% difference (95% CI, 0%-17.9%; *P* = .69) as evaluated by the MRT (Figure 2A) and 8.6% difference (95% CI, 0%-17.8%; *P* = .09) as evaluated by the AzBio Sentence Test (Figure 2B).

### Increased and Decreased Listening Effort

**Figure 3.**
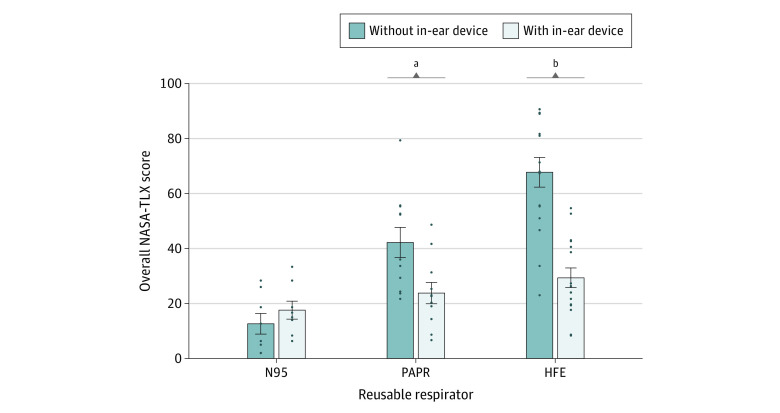
Association of In-Ear Device Use With Decreased Listening Effort While Wearing Reusable Respirators HFE indicates half-face elastomeric respirator; NASA-TLX, NASA Task Load Index; PAPR, powered air-purifying respirator. ^a^Significant at *P* = .05 threshold. ^b^Significant at *P* = .01 threshold.

Without the use of an in-ear device, participants rated the word recognition task as more effortful, as evaluated by the NASA-TLX. The mean (SD) overall workload score was 42.2 (18.2) points when the talker was using the PAPR or 67.7 (21.6) points when the talker was using the half-face elastomeric respirator compared with 12.6 (10.6) points when the N95 mask was used, the condition rated with the lowest perceived effort (Table 2). Compared with the N95 mask condition, wearing the PAPR was associated with a significant increase in overall workload score of 27.5 points (95% CI, 7.7-47.3; *P* = .001), whereas using the half-face elastomeric respirator was associated with a significant score increase of 51.7 points (95% CI, 32.7-70.6; *P* < .001) (Figure 3).

Using an in-ear device while wearing a respirator was associated with a lower listening effort, comparable to the listening effort reported with the use of the unaided N95 mask, as evaluated by the NASA-TLX. Mean (SD) overall workload was 42.2 (18.2) points while using the unaided PAPR vs 23.8 (12.8) points while using the aided PAPR, and 67.7 (21.6) points while using the unaided half-face elastomeric respirator vs 29.3 (14.4) points while using the aided half-face elastomeric respirator.

Use of an in-ear device was associated with a significantly lower overall workload score (as evaluated by the NASA-TLX) when wearing the PAPR (decrease in workload, 18.4 points; 95% CI, 0.4-36.4; *P* = .04) and an even lower score when wearing the half-face elastomeric respirator (decrease in workload, 38.4 points; 95% CI, 23.5-53.2; *P* < .001) (Figure 3). Overall workload reported in the aided PAPR condition was not significantly different from the unaided N95 mask condition (difference in workload, 9.1 points; 95% CI, −10.6 to 28.9; *P* = .93). Similarly, no significant differences were found between the aided half-face elastomeric respirator condition and the unaided N95 mask condition (difference in workload, 13.26 points; 95% CI, −5.7 to 32.2; *P* = .43) (Figure 3). These results suggest that the use of an in-ear device is a viable option for alleviating listening effort associated with the use of reusable respirators.

## Discussion

We investigated the extent to which the use of facial respirators impeded speech intelligibility and whether the use of an in-ear radio device was associated with restored communication in a realistic operating room setting. We measured speech intelligibility and cognitive load in both aided and unaided conditions using 3 types of PPE. The results indicated that an in-ear device while wearing a reusable respirator was able to restore communication to a near-baseline level in the operating room. Furthermore, testing suggested the possibility of decreasing the perceived listening effort required in the aided conditions. Therefore, use of an in-ear device presents a feasible solution that protects health care workers from airborne viral particles without impairing their communication. To our knowledge, this study is the first to propose a device-oriented solution for the current unexpected obstacle faced by health care workers. Other strategies to improve communication in the operating room include decreasing background noise, standardizing readbacks, and implementing nonverbal communication. A combination of strategies should be considered to optimize communication levels.

Impaired speech intelligibility while wearing a respirator has been described in several settings and across numerous occupations. In the law enforcement field, research by Garinther et al^10^ suggested that during gunnery tasks, as soon as speech intelligibility decreased below 93%, the wrong targets were shot. Another study noted that when speech intelligibility was 70%, as evaluated by MRT, a complex military mission would have only a 59% success rate and bear a 16% increase in performance time.^11^ Radonovich et al^2^ evaluated word intelligibility scores in health care workers using the MRT and found that when a half-face elastomeric respirator was worn, less than half of the words spoken were correctly understood, which is in line with the findings in the current study.

This study was performed in a noisy operating room setting, which is similar to that in the aforementioned studies. Use of the MRT allowed the direct comparison of these findings to results of previous work. In addition, we administered the AzBio Sentence Test to better capture the real-life speech impediment associated with reusable respirators. Overall, results of both tests demonstrated that speech intelligibility was severely impaired with use of the half-face elastomeric respirator and the PAPR but was not impaired with use of the N95 mask. Less than half of the words spoken by the talker while wearing a half-face elastomeric respirator were correctly understood by the listener in a noisy operating room. This finding is concerning because half-face elastomeric respirators are inexpensive and being used increasingly (more so than PAPRs). Some hospital systems have reduced the use of N95 masks by 75% in favor of half-face elastomeric respirators in an effort to create a long-term and cost-effective solution to the N95 mask shortage.^12^ The SonX device used in this study restored speech intelligibility to more than 90% for both the half-face elastomeric respirator and the PAPR.

We often overlook the discomfort associated with wearing a respirator. These PPEs can interfere with respiration, vision, communication, and overall well-being.^13,14^ Attempts to overcome the communication barrier by voluntarily or involuntarily (Lombard effect) increasing the voice can lead to added vocal strain, frustration, and miscommunication. Several respirator wearers may even describe increased fatigue after a full day of use. Previous research suggests that listening in difficult environments can lead to increased fatigue, stress,^15^ and even stress-related sick leave.^16^ We found that the listening effort, as evaluated by the NASA-TLX, was much higher when listening to an individual wearing a half-face elastomeric respirator or a PAPR than a regular N95 mask. Use of an in-ear device was associated with a decrease in listening effort.

### Limitations

This study has some limitations. The main limitations were the relatively small sample size and the lack of testing in real-world health care settings, which decrease the generalizability of the results. Performing a suturing task during a simulation is not as complex as performing surgery in the real world, although this fact would further indicate that the results underestimated the communication difficulties. In addition, the relatively homogeneous population does not represent the full range of personnel who are required to wear PPE in the operating room. Future studies should include more elaborate simulations, involving multiple speakers and high-stress scenarios. A randomized clinical trial in the future may examine whether these improvements in communication translate into improved surgical outcomes and reduced adverse events.

## Conclusions

This quality improvement study found that wearing reusable facial respirators impaired communication in a simulated operating room environment and that using in-ear communication devices was associated with restoration of normal communication and listening effort levels. An in-ear device was shown to be a viable, potentially life-saving solution for adequately protecting health care workers in the operating room while allowing them to communicate safely, especially during a critical time such as the COVID-19 pandemic.

## Notes

Supplement. **eTable 1.** Pairwise Comparisons for the Modified Rhyme Test (MRT) **eTable 2.** Pairwise Comparisons for AzBio Sentence Test (AzBio) **eTable 3.** Pairwise Comparisons for NASA Task Load Index (NASA-TLX) **eTable 4.** Completed Conditions for Each Participant
