# Supplementary material for: Association of In-Ear Device Use With Communication Quality Among Individuals Wearing Personal Protective Equipment in a Simulated Operating Room
Source: JAMA Netw Open. 2021 Apr 19;4(4):e216857. doi: 10.1001/jamanetworkopen.2021.6857 (PMC8056284; doi:10.1001/jamanetworkopen.2021.6857)
Supplement: Supplement. — eTable 1. Pairwise Comparisons for the Modified Rhyme Test (MRT) eTable 2. Pairwise Comparisons for AzBio Sentence Test (AzBio) eTable 3. Pairwise Comparisons for NASA Task Load Index (NASA-TLX) eTable 4. Completed Conditions for Each Participant [file jamanetwopen-e216857-s001.pdf]

Supplementary Online Content

Nguyen DL, Kay-Rivest E, Tewfik MA, Hier M, Lehmann A. Association of in-ear device use with communication quality among individuals wearing personal protective equipment in a simulated operating room. *JAMA Netw Open*. 2021;4(4):e216857. doi:10.1001/jamanetworkopen.2021.6857

eTable 1. Pairwise Comparisons for the Modified Rhyme Test (MRT)

eTable 2. Pairwise Comparisons for AzBio Sentence Test (AzBio)

eTable 3. Pairwise Comparisons for NASA Task Load Index (NASA-TLX)

eTable 4. Completed Conditions for Each Participant

This supplementary material has been provided by the authors to give readers additional information about their work.

© 2021 Nguyen DL et al. *JAMA Network Open.*

eTable 1. Pairwise Comparisons for the Modified Rhyme Test (MRT)

	Score Difference, % points	95% Confidence Interval	P-value
HFE, Unaided – N95, Unaided	-44.1	-55.7 to -33.1	<0.001
HFE, Unaided – PAPR, Unaided	-26.2	-37.0 to -15.4	<0.001
HFE, Unaided – HFE, Aided	-37.7	-47.3 to -28.1	<0.001
HFE, Unaided – N95, Aided	-43.3	-54.6 to -32.0	<0.001
HFE, Unaided – PAPR, Aided	-41.2	-52.0 to -30.4	<0.001
N95, Unaided – PAPR, Unaided	18.2	6.0 to 30.3	<0.001
N95, Unaided – HFE, Aided	6.7	-4.6 to 17.9	0.691
N95, Unaided – N95, Aided	1.1	-11.2 to 13.5	1.000
N95, Unaided – PAPR, Aided	3.2	-8.9 to 15.3	1.000
PAPR, Unaided – HFE, Aided	-11.5	-22.3 to -0.7	0.028
PAPR, Unaided – N95, Aided	-17.1	-29.2 to -4.9	0.001
PAPR, Unaided – PAPR, Aided	-15.0	-26.7 to -3.3	0.004
HFE, Aided – N95, Aided	-5.6	-16.8 to 5.7	0.890
HFE, Aided – PAPR, Aided	-3.5	-14.3 to 7.3	0.998
N95, Aided – PAPR, Aided	2.1	-10.1 to 14.2	1.000

Abbreviations: PAPR, Powered Air Purifying Respirator; HFE, half-face elastomeric. Unaided: Tested without the use of the in-ear communication device. Aided: Tested with the use of the in-ear communication device.

© 2021 Nguyen DL et al. *JAMA Network Open.*

eTable 2. Pairwise Comparisons for AzBio Sentence Test (AzBio)

	Score Difference, % points	95% Confidence Interval	P-value
HFE, Unaided – N95, Unaided	-40.9	-50.1 to -31.6	<0.001
HFE, Unaided – PAPR, Unaided	-26.1	-34.5 to -17.6	<0.001
HFE, Unaided – HFE, Aided	-32.3	-40.7 to -23.8	<0.001
HFE, Unaided – N95, Aided	-36.3	-45.6 to -27.1	<0.001
HFE, Unaided – PAPR, Aided	-36.0	-44.4 to -27.5	<0.001
N95, Unaided – PAPR, Unaided	14.8	5.5 to 24.0	<0.001
N95, Unaided – HFE, Aided	8.6	-0.7 to 17.8	0.090
N95, Unaided – N95, Aided	4.5	-5.2 to 14.3	0.924
N95, Unaided – PAPR, Aided	4.9	-4.3 to 14.2	0.823
PAPR, Unaided – HFE, Aided	-6.2	-14.7 to 2.3	0.351
PAPR, Unaided – N95, Aided	-10.2	-19.5 to -1.0	0.020
PAPR, Unaided – PAPR, Aided	-9.9	-18.3 to -1.4	0.011
HFE, Aided – N95, Aided	-4.0	-13.3 to 5.2	0.954
HFE, Aided – PAPR, Aided	-3.7	-12.1 to 4.8	0.955
N95, Aided – PAPR, Aided	0.4	-8.9 to 9.6	1.000

Abbreviations: PAPR, Powered Air Purifying Respirator; HFE, half-face elastomeric. Unaided: Tested without the use of the in-ear communication device. Aided: Tested with the use of the in-ear communication device.

© 2021 Nguyen DL et al. *JAMA Network Open.*

eTable 3. Pairwise Comparisons for NASA Task Load Index (NASA-TLX)

	Overall Workload Difference	95% Confidence Interval	P-value
HFE, Unaided – N95, Unaided	51.7	32.7 to 70.6	<0.001
HFE, Unaided – PAPR, Unaided	24.2	7.4 to 40.9	<0.001
HFE, Unaided – HFE, Aided	38.4	23.5 to 53.3	<0.001
HFE, Unaided – N95, Aided	46.7	27.8 to 65.7	<0.001
HFE, Unaided – PAPR, Aided	42.6	25.8 to 59.3	<0.001
N95, Unaided – PAPR, Unaided	-27.5	-47.3 to -7.7	0.001
N95, Unaided – HFE, Aided	-13.3	-32.2 to 5.7	0.427
N95, Unaided – N95, Aided	-5.0	-26.0 to 16.1	1.000
N95, Unaided – PAPR, Aided	-9.1	-28.9 to 10.7	0.932
PAPR, Unaided – HFE, Aided	14.2	-2.5 to 31.0	0.164
PAPR, Unaided – N95, Aided	22.6	2.8 to 42.3	0.015
PAPR, Unaided – PAPR, Aided	18.4	0.4 to 36.4	0.041
HFE, Aided – N95, Aided	8.3	-10.6 to 27.3	0.953
HFE, Aided – PAPR, Aided	4.2	-12.6 to 20.9	1.000
N95, Aided – PAPR, Aided	-4.2	-23.9 to 15.6	1.000

Abbreviations: PAPR, Powered Air Purifying Respirator; HFE, half-face elastomeric. Unaided: Tested without the use of the in-ear communication device. Aided: Tested with the use of the in-ear communication device.

© 2021 Nguyen DL et al. *JAMA Network Open.*

eTable 4. Completed Conditions for Each Participant

	MRT			AzBio			NASA-TLX		
	N95	PAPR	HFE	N95	PAPR	HFE	N95	PAPR	HFE
Participant ID									
01	X		X	X	X	X	X		X
02			X		X	X			X
03		X	X		X	X		X	X
04		X	X		X	X		X	X
05	X	X	X	X	X	X	X	X	X
06	X	X	X	X	X	X	X	X	X
07	X	X	X	X	X	X	X	X	X
08	X	X	X	X	X	X	X	X	X
09	X	X	X	X	X	X	X	X	X
10	X	X	X	X	X	X	X	X	X
11	X	X	X	X	X	X	X	X	X
12	X	X	X	X	X	X	X	X	X
n =	9	10	12	9	12	12	9	10	12

Abbreviations: MRT, modified rhyme test; AzBio, AzBio sentence test; NASA-TLX, NASA task load index; PAPR, Powered Air Purifying Respirator; HFE, half-face elastomeric

© 2021 Nguyen DL et al. *JAMA Network Open.*
